# In memoriam: Davise Honig Larone

**DOI:** 10.1128/jcm.01844-25

**Published:** 2026-03-18

**Authors:** Kathy Fauntleroy, Ellen Jo Baron, Romney M. Humphries

**Affiliations:** 1 NewYork-Presbyterian/Weill Cornell Medical Center 159947 , New York, New York, USA; 2 Department of Pathology (Emerita), Stanford University School of Medicine 10624 , Stanford, California, USA; 3 Vanderbilt University Medical Center 12328, Nashville, Tennessee, USA; Vanderbilt University Medical Center, Nashville, Tennessee, USA

**Keywords:** Davise Larone, obituary, mycology

## EDITORIAL

On 6 September 2025, we lost a beloved and influential microbiologist, Davise Honig Larone, PhD, D(ABMM). Davise’s legacy is apparent in any clinical mycology laboratory and training program in the United States, where you are likely to find a copy of her influential text, *Medically Important Fungi: A Guide to Identification*. Chock-full of practical guidance and beautifully hand-illustrated by Davise, this textbook has become the on-the-bench field guide to morphological identification of fungi. In person, Davise was brilliant and hilarious and demonstrated tremendous kindness and mentorship to many of us in the clinical microbiology field. We will be forever indebted to her contributions. In this article, the authors provide a tribute to this legend in our field.

**Figure:**
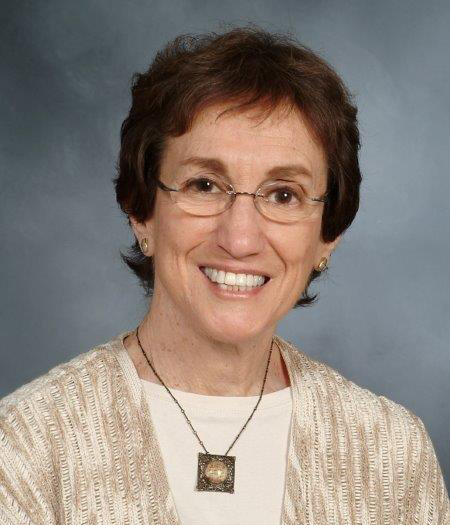


## IN MEMORIAM

One of the most beloved and influential microbiologists of her generation, Davise Honig Larone, PhD, D(ABMM), professor emerita at Weill Cornell Medical College, passed away peacefully on 6 September 2025, at the age of 86.

Dr. Larone, a world-renowned clinical microbiologist, is best known for creating, authoring, and illustrating *Medically Important Fungi: A Guide to Identification*, the gold standard diagnostic guide and most widely referred to medical mycology resource for fungal identification among clinical laboratory scientists worldwide.

A long-time member of the American Society for Microbiology, Dr. Larone served on the editorial board of the *Journal of Clinical Microbiology* and as executive council and program chair of the New York City Branch of the ASM. She was a Diplomate of the American Board of Medical Microbiology and the American Board of Medical Laboratory Immunology, a member of several medical mycology societies, and a Fellow of the American Academy of Microbiology.

Dr. Larone’s accomplishments are many, but it was her character, one of authenticity, humor, and engaging teaching style, that set her apart. At an ASM meeting held in San Diego on 31 October (Halloween), after her introduction as the keynote speaker, Dr. Larone pulled an enormous black peaked hat from beneath the podium, plopped it on her head, and announced, “I come to you from New York City on this Halloween morning as the Wicked Witch of the YEAST!” The crowd erupted in laughter, and the lecture on fungal identification proceeded with a fully captivated audience.

Along with the esteem of microbiologists everywhere, Dr. Larone received numerous awards for teaching and contributions to clinical mycology, including the Billy H. Cooper Memorial Award in 2005 from the Medical Mycology Society of the Americas, and the Max Littman Award in 2008, for contributions to medical mycology by the Medical Mycology Society of New York. In 2014, Dr. Larone received the career pinnacle, ASM bioMérieux Sonnenwirth Award for Leadership in Clinical Microbiology, honoring her lifetime of impactful achievements.

Born in New York City on 28 June 1939, Davise Larone, daughter of Sidney Honig and Rose Kahn Honig and sister of Marvin Honig, in her youth moved with her family to Louisville, Kentucky, where she attended the University of Louisville, studying medical laboratory technology before beginning her career in 1960 at the University of Cincinnati as a medical laboratory scientist. Davise traveled abroad, working for 2 years in immunology at Tel Aviv University. Once back in the United States, she earned her master’s degree in biology and education from Long Island University, C.W. Post Center, and a PhD from New York University in 1985. During this time, Davise raised her young daughter, Ronit, while also working full time at Lenox Hill Hospital, rising from technologist to Chief of Microbiology. In 1997, she joined NewYork-Presbyterian/Weill Cornell Medical Center as Director of the Clinical Microbiology Laboratory and simultaneously joined the faculty at Weill Cornell Medical College, receiving her professor emerita title in 2012.

Davise’s late husband, John D. Lawrence, was a biomedical publisher whose lasting work included introducing the comprehensive Clinical Microbiology Newsletter, published now for over 45 years.

Throughout her distinguished career, Dr. Larone remained a medical laboratory scientist at heart and also an artist. Early on, she combined her desire for teaching herself how to identify organisms with her passion for drawing. This led to Dr. Larone’s unique practice of intricately illustrating what she saw microscopically, an approach that ultimately developed into the first edition of her groundbreaking publication, *Medically Important Fungi: A Guide to Identification*, which she wrote and illustrated as a clear identification tool for clinical laboratory personnel as well as for students of medical technology and mycology. Today*,* nearly a half century since its origination, the book has been translated into other languages and now, in its 7th edition, bears her name *Larone’s Medically Important Fungi: A Guide to Identification*.

Dr. Larone presented more than 100 workshops and lectures in 52 cities across the United States and in 14 cities in 9 other countries. When attending conferences and giving lectures, microbiologists from hospitals worldwide would approach Davise, expressing how invaluable and well worn their copies of her book were from much use at the bench in the laboratory. The bench was exactly where she wanted her book to be. She was passionate about learning and just as passionate about sharing her learnings with others. *Larone’s Medically Important Fungi: A Guide to Identification* is the embodiment of that legacy for microbiologists around the world.

Greatly respected by her colleagues for her kindness, dedication, wisdom, and abounding energy, Dr. Larone will forever be remembered for her caring mentorship of staff and students. She will be deeply missed. She is survived by her daughter Ronit; granddaughter Jessica, whom she treasured; stepdaughter Elizabeth and all of Davise’s loving family and cherished friends.

